# Association of the Apolipoprotein B/Apolipoprotein A-I Ratio, Metabolic Syndrome Components, Total Cholesterol, and Low-Density Lipoprotein Cholesterol with Insulin Resistance in the Population of Georgia

**DOI:** 10.1155/2014/925650

**Published:** 2014-05-13

**Authors:** Zaza Makaridze, Elene Giorgadze, Ketevan Asatiani

**Affiliations:** ^1^“Medicore” Ltd. Tbilisi State Medical University Affiliated Clinic, 0186 Tbilisi, Georgia; ^2^National Institute of Endocrinology, Tbilisi State University Affiliated Clinic, 0159 Tbilisi, Georgia

## Abstract

The study was designed to assess the association between insulin resistance (IR) and apolipoprotein B/apolipoprotein A-I ratio (ApoB/ApoA-I ratio), metabolic syndrome (MetS) components, total cholesterol (TC), and low-density lipoprotein cholesterol (LDL-C) in the nondiabetic population of Georgia. The subjects were 1522 Georgians of Caucasian origin (mean age = 45 years, 653 women) without diabetes who had visited the clinics for a related health checkup between 2012 and 2013. IR was calculated using the computer homeostasis model assessment (HOMA2-IR) and was defined as the upper quartile. MetS was diagnosed using the updated ATP-III definition of the metabolic syndrome. Logistic and multiple regression models were used to estimate the association between IR and other components. IR was positively correlated with age, ApoB, ApoB/ApoA-I ratio, MetS components (excluding high-density lipoprotein cholesterol—HDL-C), LDL-C, fasting insulin, and TC and negatively correlated with HDL-C and ApoA-I in both sexes (all *P* < 0.001). In the logistic regression models, gender, age, ApoB/ApoA-I ratio, diastolic pressure, HDL-C, LDL-C, fasting glucose, and triglycerides were the covariates significantly associated with IR (OR: 8.64, 1.03, 17.95, 1.06, 0.13, 1.17, 3.75, and 2.29, resp.; all *P* < 0.05). Multiple regression models demonstrated that these components (except for HDL-C) made an independent contribution to the prediction of HOMA2 (all *P* < 0.05).

## 1. Introduction

Insulin resistance (IR) is a metabolic disorder independently associated with cardiovascular disease [1–3]. IR is associated with aging and a cluster of important cardiometabolic risk factors (dyslipidemia, arterial hypertension, hyperglycemia, and obesity) and is believed to be the common shared pathophysiological disturbance [4–8]. Thus, accurate and early prediction and detection of IR are very important in clinical practice so as to identify patients at high risk for cardiovascular disorders. Evaluation and revealing of the potential additional metabolic markers can help predict cardiovascular risk better.

Prospective risk studies, such as AMORIS [9–11], INTERHEART [12], EPIC-Norfolk study [13, 14], and ULSAM [15], indicate that apolipoprotein B/apolipoprotein A-I ratio (ApoB/ApoA-I ratio) is a strong predictor of risk of myocardial infarction (MI). A recent review of existing evidence by Walldius supports the use of ApoB/ApoA-I ratio as a strong predictor of cardiovascular risk [16].

Some studies have indicated that IR was significantly associated with ApoB/ApoA-I ratio, metabolic syndrome (MetS), and lipid indices. Sierra-Johnson et al. studied 2.955 adults (mean age 47 years, 1.457 women) without diabetes from the US NHANS III population. The ApoB/ApoA-I ratio was an independent predictor of insulin resistance after adjustment for age and race and remained significant after further adjustment for MetS components and traditional and inflammatory risk factors [17]. Ying et al. investigated 3.945 men and 2.141 women with abdominal obesity. Both low-density lipoprotein cholesterol (LDL-C) and ApoB/ApoA-I ratio were independent risk factors for Mets, and ApoB/ApoA-I ratio was stronger in this regard for obese population [18].

There is no Georgian report available about this subject. The aim of our study was to assess the association between insulin resistance and apolipoprotein B/apolipoprotein A-I ratio, MetS components, total cholesterol (TC), and low-density lipoprotein cholesterol (LDL-C) in the population of Georgia. We also analyzed the independence of these associations.

## 2. Materials and Methods

### 2.1. Subjects

We analyzed 1522 Georgians of Caucasian origin, who had visited the National Institute of Endocrinology (Tbilisi State University Affiliated Clinic) and “Medicore” Ltd. (Tbilisi State Medical University Affiliated Clinic) for a related health checkup between 2012 and 2013. Subjects were men and nonpregnant women aged ≥18 years and <80 years (653 women and 869 men), who attended the morning medical examination and who had fasted ≥8 h. Exclusion criteria used were missing readings for ApoB, ApoA-I, MetS components, low-density lipoprotein cholesterol (LDL-C), total cholesterol (TC), and all self-reported diabetics and/or subjects on medication for this disease and/or those with fasting glucose ≥7 mmol/l (126 mg/dL) and/or postprandial glucose ≥11.1 mmol/l (200 mg/dL).

### 2.2. Anthropometric Measurements and Laboratory Data

Height and weight were obtained using standardized techniques and equipment. Waist circumference (WC) was measured in the erect position with a soft tape at the middle between the lowest rib and the iliac crest. Body mass index (BMI) was calculated as weight (kg)/(height[m])^2^.

Systolic blood pressure (SP) and diastolic blood pressure (DP) were measured twice in the sitting position after at least 15 minutes of rest. The mean of the two measurements was used in the analyses. Insulin sensitivity index was determined using the updated computer homeostasis model assessment (HOMA2-IR) index. Venous blood samples were drawn after a minimum of 8 h of fasting. ApoB and ApoA-I levels were measured by the immunoturbidimetric assay with Roche Diagnostics kit, using Roche/Hitachi C311 analyzer. Glucose was measured after enzymatic oxidation in the presence of glucose oxidase with HUMAN Diagnostics kit (Germany), using HUMALYZER 2000 analyzer. Total cholesterol (TC) was measured after enzymatic hydrolysis and oxidation with enzymatic colorimetric test for cholesterol with lipid clearing factor (HUMAN Diagnostics kit, Germany), using HUMALYZER 2000 analyzer. High-density cholesterol lipoprotein (HDL-C) was measured in the supernatant after precipitating the other lipoproteins with phosphotungstic acid and magnesium chloride and centrifugation with HUMAN Diagnostics kit (Germany), using HUMALYZER 2000 analyzer. The triglycerides (TG) were measured after enzymatic hydrolysis with lipases by enzymatic colorimetric test for triglycerides with lipid clearing factor (HUMAN Diagnostics kit, Germany), using HUMALYZER 2000 analyzer. LDL-C was calculated by Friedewald formula. When TG level was greater than or equal to 200 mg/dL (2.3 mmol/l), LDL-C level was measured by direct method with HUMALYZER 2000 analyzer. Insulin was measured by ELISA method with HUMAN Diagnostics kit (Germany), using ELISYS UNO analyzer.

### 2.3. Metabolic Syndrome Definition

The updated ATP-III definition of MetS [19] was used when any three or more criteria were present: waist circumference (WC) ≥ 102 cm in men and ≥88 cm in women; impaired fasting glucose-fasting blood glucose (FG) of ≥5.6 mmol/l (100 mg/dL); systolic blood pressure (SP) ≥ 130 mmHg and/or diastolic blood pressure (DP) ≥ 85 mmHg or treatment of previously diagnosed hypertension; fasting levels of triglycerides (TG) ≥ 1.7 mmol/l (150 mg/dL) or treatment for this abnormality; fasting high-density lipoprotein cholesterol (HDL-C) < 1.03 mmol/l (40 mg/dL) for men and <1.30 mmol/l (50 mg/dL) for women or treatment for this abnormality.

### 2.4. Arterial Hypertension Definition

Subjects were considered having hypertension if they were taking antihypertensive medications and/or having systolic blood pressure ≥ 140 mmHg and/or having diastolic blood pressure ≥ 90 mmHg, as defined by the recent Joint National Committee on Prevention, Detection, Evaluation, and Treatment of High Blood Pressure guidelines.

### 2.5. Dyslipidemia Definition

Subjects were considered to have dyslipidemia if they had fasting levels of triglycerides (TG) ≥ 1.7 mmol/l (150 mg/dL) and/or fasting levels of high-density lipoprotein cholesterol (HDL-C) < 1.03 mmol/l (40 mg/dL) in men and <1.30 mmol/l (50 mg/dL) in women and/or fasting level of total cholesterol (TC) ≥ 5.2 mmol/l (200 mg/dL) and/or fasting level of low-density lipoprotein cholesterol (LDL-C) ≥ 4.10 mmol/l (160 mg/dL) and/or used medications for this abnormality.

### 2.6. Statistical Analysis

All the analyses were performed using IBM SPSS Statistics for Windows (version 22.0).

IR was defined as the gender-specific upper quartile of HOMA2-IR and insulin sensitivity (IS) as the remaining three quartiles. We used the Mann-Whitney *U* test to compare related data between sexes and between IR and IS subjects. Chi-square test was used to compare the prevalence of MetS, AH, and dyslipidemia between sexes and between IR and IS subjects.

Spearman partial correlation analysis of HOMA2-IR with ApoB/ApoA-I ratio, MetS components, BMI, TC, and LDL-C in men and women (controlling by age) was done. Further partial correlation analysis of ApoB/ApoA-I ratio with HOMA2-IR in both sexes was performed, after adjustment for age, MetS components, BMI, TC, and LDL-C.

A logistic regression was carried out to assess the association between IR and ApoB/ApoA-I ratio, MetS components, and LDL-C. Because of high correlation of HOMA2-IR with TC (*r* = 0.73), WC (*r* = 0.78), and SP (*r* = 0.72), these variables were not included into the regression models. Selection of predictor variables was done by entry (blockwise selection) method. To analyze the additional contribution of ApoB/ApoA-I and MetS components to IR, multiple linear regression was used (stepwise method). The predictive value of each variable was assessed by comparing *R* ^2^ values obtained from each model.

## 3. Results

Descriptive characteristics between sexes are shown in Table 1.

The ApoB/ApoA-I ratio, ApoB, blood pressure levels, WC, BMI, LDL-C, FG, FIns, TG, TC, HOMA2-IR, the prevalence of MetS, AH, and dyslipidemia were all significantly higher but HDL-C and ApoA-I levels were significantly lower in men than in women (all *P* < .0001).

Therefore, we stratified the analysis by sex.

Insulin resistant subjects in both sexes were older and had greater mean values for ApoB/ApoA-I ratio, ApoB, blood pressure levels, WC, BMI, LDL-C, FG, FIns, TG, TC, and HOMA2-IR than insulin sensitive subjects. On the contrary, IR subjects had significantly lower HDL-C and ApoA-I levels than IS subjects (all *P* < .0001). The prevalence of MetS, AH, and dyslipidemia was significantly higher in IR subjects (all *P* < .0001) (Tables 2 and 3).

ApoB level, ApoB/ApoA-I ratio, and LDL-C level increased significantly and gradually among HOMA2-IR quartiles (Table 4).

**Figure 1:**
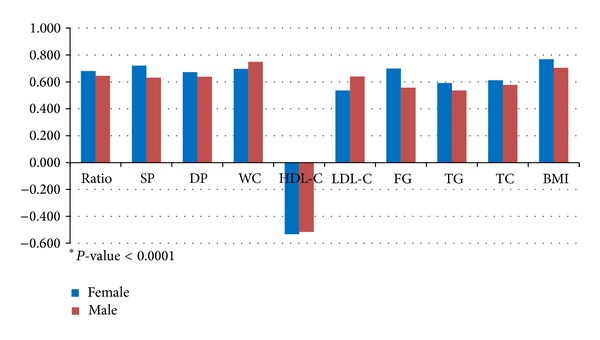
Spearman partial correlation (*r*) analysis of HOMA2-IR with ApoB/ApoA-I ratio, MetS components, BMI (body mass index), TC, and LDL-C in men and women (controlling by age). Partial correlation: Spearman partial correlation coefficient *r*; *P* (2-tailed) <.0001. ApoB/ApoA-I ratio: apolipoprotein B/apolipoprotein A-I ratio, SP: systolic blood pressure, DP: diastolic blood pressure, WC: waist circumference, BMI: body mass index, HDL-C: high-density lipoprotein cholesterol, LDL-C: low-density lipoprotein cholesterol, FG: fasting glucose, TG: triglyceride, TC: total cholesterol, and HOMA2-IR: homeostasis model assessment of insulin resistance.

**Figure 2:**
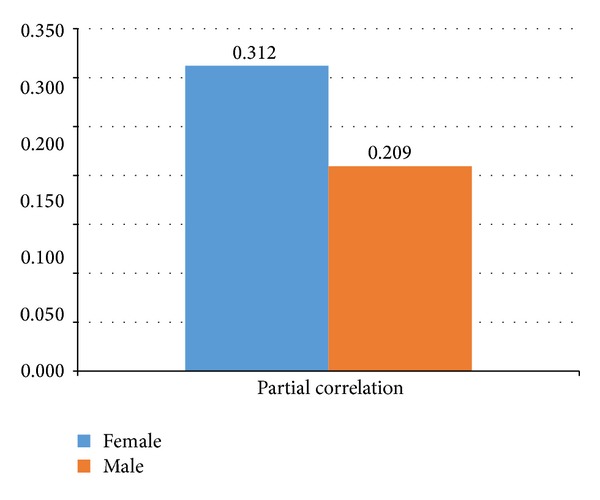
Partial correlation (*r*) of ApoB/ApoA-I ratio with HOMA2-IR (controlling by age, SP, DP, WC, BMI, HDL-C, LDL-C, FG, TG, and TC). Partial correlation: Spearman partial correlation coefficient *r*; *P* (2-tailed) <.0001. ApoB/ApoA-I ratio: apolipoprotein B/apolipoprotein A-I ratio, IR: insulin resistance, SP: systolic blood pressure, DP: diastolic blood pressure, WC: waist circumference, BMI: body mass index, HDL-C: high-density lipoprotein cholesterol, LDL-C: low-density lipoprotein cholesterol, FG: fasting glucose, TG: triglyceride, TC: total cholesterol, and HOMA2-IR: homeostasis model assessment of insulin resistance.

Results of spearman partial correlation analysis of HOMA2-IR with ApoB/ApoA-I ratio, MetS components, BMI, TC, and LDL-C in men and women are shown in Figure 1. Further partial correlation of ApoB/ApoA-I ratio with IR in men and women was done (Figure 2). There was still significant positive correlation between them (*r* = 0.312 for women and 0.209 for men, both *P* < .0001).

Logistic regression models demonstrated that gender, age, ApoB/ApoA-I ratio, diastolic blood pressure, HDL-C, LDL-C, fasting glucose, and triglycerides were the covariates significantly associated with the presence of IR (OR: 8.64, 1.03, 17.95, 1.06, 0.13, 1.17, 3.75, and 2.29, resp.; all *P* < .05) (Table 5).

Multiple regression models demonstrated that ApoB/ApoA-I ratio, FG, DP, gender, LDL-C, age, and TG made a significant and independent contribution to the prediction of HOMA2-IR (*R* ^2^ change: 0.421, 0.133, 0.039, 0.026, 0.014, 0.004, and 0.002, resp.; all *P* < .05), with ApoB/ApoA-I ratio being the strongest predictor (Table 6). HDL-C was considered nonsignificant.

## 4. Discussion

ApoB and ApoA-I are the two major apolipoproteins involved in lipid transport and in the processes causing atherosclerosis and its complications [16]. ApoB is the major protein in very low-density lipoproteins (VLDL), intermediate-density lipoproteins (IDL), and low-density lipoproteins (LDL), one protein per particle [20]. ApoA-I is the major protein in high-density lipoprotein (HDL) particles. The ApoB number indicates the total number of atherogenic particles; the higher the number the higher the cardiovascular (CV) risk. ApoA-I reflects the antiatherogenic potential in HDL particles; the higher the value the better the protection against CV risk. The ApoB/ApoA-I ratio indicates the balance between atherogenic and antiatherogenic particles; the higher the value, the higher the CV risk.

Many authors have reviewed the importance of ApoB, ApoA-I, and ApoB/ApoA-I ratio as markers of atherogenic risk [9, 11, 21–27]. Prospective risk studies, such as AMORIS [9–11], INTERHEART [12], EPIC-Norfolk study [13, 14], and ULSAM [15], indicate that ApoB/ApoA-I ratio is a very useful predictor of risk of myocardial infarction (MI). In the AMORIS study it was found that ApoB was significantly better to predict risk than LDL-C, especially for those with low values of LDL-C, and ApoB/ApoA-I ratio had a significantly stronger relation with MI than any other lipid-based ratio. INTERHEART case-control study showed that, in all 52 countries investigated, ApoB/ApoA-I ratio was not only the strongest factor in explaining risk of acute MI, but also the most prevalent risk factor of all the nine conventional risk factors investigated irrespective of age, sex, race, and other lipids or lipid ratios. In the ULSAM study a risk prediction score for MI including proinsulin and the ApoB/ApoA-I ratio was developed in middle-aged men. This score was highly predictive for future fatal and nonfatal MI and proved to be at least as good as the Framingham and the PROCAM scores.

The prevalence of IR is increasing worldwide [28–31]. Studies have indicated that IR was significantly associated with ApoB/ApoA-I ratio, metabolic syndrome (MetS), and lipid indices. Sierra-Johnson et al. studied 2.955 adults (mean age 47 years; 1.457 women) without diabetes from the US NHANS III population. The ApoB/ApoA-I ratio was an independent predictor of insulin resistance after adjustment for age and race and remained significant after further adjustment for MetS components and traditional and inflammatory risk factors [17]. Similar results were found in our study.

Ying et al. investigated 3.945 men and 2.141 women with abdominal obesity. The study showed that the correlation coefficient between ApoB/ApoA-I ratio and HOMA2-IR was higher than between LDL-C and HOMA2-IR in both sexes (with more differences in females), as it was found in our study. Both LDL-C and ApoB/ApoA-I ratio were independent risk factors for MetS, and ApoB/ApoA-I ratio was stronger in this regard for obese population [18].

Belfki et al. have shown in a Tunisian population (330 adults aged 35–74) that ApoB/ApoA-I ratio was strongly associated with MetS, with each of the components, and with increasing number of components, as well as with IR [32]. Another study reported that subjects with higher levels of ApoB had a higher proportion of the MetS components in comparison with patients having higher LDL-C and normal level of ApoB [33]. Some studies have shown that measurement of the ApoB/ApoA-I ratio may improve vascular risk prediction. Higher ApoB/ApoA-I ratio has been associated with increased cardiac events [24].

Head to head analysis (Table 4) of ApoB, ApoB/ApoA-I ratio, and LDL-C among HOMA2-IR quartiles showed that in IR subjects emphasis in risk evaluation and therapy should be made on ApoB and ApoB/ApoA-I ratio rather than on LDL-C, because LDL-C level might be in “optimal” or “near optimal” range. The study showed the possibility of focusing patients at “high risk” of having IR according to ApoB/ApoA-I ratio (≥1.12 in men and ≥1.0 in women, *P* < .0001). These findings correlate well with other authors [18, 34]. Carnevale et al. enrolled 616 patients with normal glucose tolerance (NGT) and found that, in NGT with LDL-C < 100 mg/dL, a higher ApoB/ApoA-I exhibited an atherogenic lipid profile, indicating that LDL-C alone is insufficient to define CV risk. Independent from LDL-level, when ApoB/ApoA-I is lower, the lipid profile is, in fact, less atherogenic. This study demonstrates that ApoB/ApoA-I is at least complementary to LDL-C in identifying the “effective” CV risk profile of asymptomatic NGT subjects [34].

Enkhmaa et al. have studied several ethnic groups of European and African Americans and developed a CV risk score which was found to be significantly increased across tertiles of ApoB/ApoA-I ratio. They concluded that ApoB/ApoA-I ratio differed across ethnicities and was associated with the presence of MetS in both groups. Among African Americans, an elevated ApoB/ApoA-I ratio independently predicted a greater risk of CAD [35].

Based on these findings, Sniderman and Faraj recommended that the dyslipidemia be redefined to include ApoB and ApoA-I as stronger risk marker especially compared to LDL-C (often low in MetS), TG, and HDL-C [36].

This study has several limitations. Calculated HOMA index is well, but not perfectly correlated with values of insulin sensitivity from the euglycemic clamp, which is considered the gold standard for measurement of insulin sensitivity. Another limitation is that the subjects were not a general population but visitors to the clinic.

## 5. Conclusion

ApoB/ApoA-I ratio, MetS components, LDL-C, TC, gender, and age were all found to be associated with IR in the Georgian population. But ApoB/ApoA-I ratio was stronger in this regard, highlighting the fact that in IR patients emphasis in risk evaluation and therapy should be made on ApoB/ApoA-I ratio rather than on LDL-C level. The independent and significant association of the ApoB/ApoA-I ratio with IR demonstrates the usefulness of using this ratio as a valuable predictor of IR and an important risk factor in the cardiovascular risk assessment.

## Figures and Tables

**Table 1:** Descriptive characteristics between sexes.

	Pooled (*n* = 1522)	Female (*n* = 653; 43%)	Male (*n* = 869; 57%)	*P* value
Age	45 ± 18	46 ± 18	44 ± 17	.023
ApoB/ApoA-I ratio	0.79 ± 0.3	0.71 ± 0.3	0.85 ± 0.3	<.0001
ApoA-I (g/L)	1.06 ± 0.42	1.07 ± 0.39	1.06 ± 0.44	<.0001
ApoB (g/L)	0.81 ± 0.34	0.74 ± 0.31	0.86 ± 0.34	<.0001
SP (mmHg)	134 ± 26	123 ± 19	142 ± 28	<.0001
DP (mmHg)	84 ± 15	77 ± 11	89 ± 16	<.0001
WC (cm)	88 ± 14	83 ± 13	92 ± 13	<.0001
BMI (kg/m^2^)	27 ± 7	25 ± 5.5	28 ± 7	<.0001
HDL-C (mmol/L)	1.28 ± 0.3	1.41 ± 0.2	1.19 ± 0.2	<.0001
LDL-C (mmol/L)	2.92 ± 0.7	2.74 ± 0.5	3.06 ± 0.7	<.0001
FG (mmol/L)	5.42 ± 0.8	5.33 ± 0.9	5.49 ± 0.7	<.0001
FIns (*μ*U/mL)	14.66 ± 10.5	13.75 ± 11.17	15.35 ± 9.94	<.0001
TG (mmol/L)	1.89 ± 0.4	1.73 ± 0.3	2.0 ± 0.5	<.0001
TC (mmol/L)	4.70 ± 0.8	4.46 ± 0.7	4.88 ± 0.8	<.0001
HOMA2-IR	1.57 ± 0.50	1.87 ± 1.36	2.04 ± 1.24	<.0001
MetS *n* (%)	552 (36.3)	190 (29.1)	362 (41.7)	<.0001
AH *n* (%)	742 (48.8)	185 (28.3)	557 (64.1)	<.0001
Dyslipidemia *n* (%)	783 (51.4)	265 (40.6)	518 (59.6)	<.0001

Data are expressed as mean ± SD.

The Mann-Whitney *U* test was used to compare the data between sexes.

Chi-square test was used to compare the prevalence of MetS, AH, and dyslipidemia between sexes.

ApoB/ApoA-I ratio: apolipoprotein B/apolipoprotein A-I ratio, ApoA-I: apolipoprotein A-I, ApoB: apolipoprotein B, SP: systolic blood pressure, DP: diastolic blood pressure, WC: waist circumference, BMI: body mass index, HDL-C: high-density lipoprotein cholesterol, LDL-C: low-density lipoprotein cholesterol, FG: fasting glucose, FIns: fasting insulin, TG: triglyceride, TC: total cholesterol, HOMA2-IR: homeostasis model assessment of insulin resistance, MetS: metabolic syndrome, and AH: arterial hypertension.

**Table 2:** Descriptive characteristics between insulin resistant and insulin sensitive (women).

	Insulin sensitive	Insulin resistant	*P* value
Women *n* (%)	491 (75.2)	162 (24.8)	
Age	45.5 ± 18.25	50.95 ± 15.07	<.0001
ApoB/ApoA-I ratio	0.62 ± 0.17	1.0 ± 0.23	<.0001
ApoA-I (g/L)	1.08 ± 0.35	1.06 ± 0.47	<.0001
ApoB (g/L)	0.65 ± 0.22	1.01 ± 0.39	<.0001
SP (mmHg)	115 ± 12.4	144 ± 18.38	<.0001
DP (mmHg)	74 ± 8.28	89 ± 11.06	<.0001
WC (cm)	79 ± 10.36	98 ± 7.75	<.0001
BMI (kg/m^2^)	23 ± 4.04	34.05 ± 5.48	<.0001
HDL-C (mmol/L)	1.47 ± 0.20	1.20 ± 0.18	<.0001
LDL-C (mmol/L)	2.62 ± 0.38	3.09 ± 0.63	<.0001
FG (mmol/L)	5.0 ± 0.71	6.30 ± 0.53	<.0001
FIns (*μ*U/mL)	8.24 ± 3.31	30.44 ± 9.94	<.0001
Tryg (mmol/L)	1.63 ± 0.15	1.97 ± 0.37	<.0001
TC (mmol/L)	4.20 ± 0.59	5.24 ± 0.55	<.0001
MetS *n* (%)	49 (10)	141 (87)	<.0001
AH *n* (%)	63 (12.8)	122 (75.3)	<.0001
Dyslipidemia *n* (%)	110 (22.4)	155 (95.7)	<.0001

Data are expressed as mean ± SD.

The Mann-Whitney *U* test was used to compare the data between insulin sensitive and insulin resistant.

Chi-square test was used to compare the prevalence of MetS, AH, and dyslipidemia between insulin sensitive and insulin resistant.

ApoB/ApoA-I ratio: apolipoprotein B/apolipoprotein A-I ratio, ApoA-I: apolipoprotein A-I, ApoB: apolipoprotein B, SP: systolic blood pressure, DP: diastolic blood pressure, WC: waist circumference, BMI: body mass index, HDL-C: high-density lipoprotein cholesterol, LDL-C: low-density lipoprotein cholesterol, FG: fasting glucose, FIns: fasting insulin, TG: triglyceride, TC: total cholesterol, HOMA2-IR: homeostasis model assessment of insulin resistance, MetS: metabolic syndrome, and AH: arterial hypertension.

Insulin sensitive (defined as the gender-specific first three quartiles) HOMA2-IR (homeostasis model assessment of insulin resistance) <2.3; insulin resistant (defined as the gender-specific upper quartile) HOMA2-IR (homeostasis model assessment of insulin resistance) ≥2.3.

**Table 3:** Descriptive characteristics between insulin resistant and insulin sensitive (men).

	Insulin sensitive	Insulin resistant	*P* value
Men *n* (%)	659 (75.8)	210 (24.2)	
Age	42.4 ± 17.6	52 ± 14.65	<.0001
ApoB/ApoA-I ratio	0.76 ± 0.19	1.12 ± 0.32	<.0001
ApoA-I (g/L)	1.05 ± 0.42	1.04 ± 0.30	<.0001
ApoB (g/L)	0.78 ± 0.30	1.11 ± 0.36	<.0001
SP (mmHg)	133 ± 21.77	171 ± 23.26	<.0001
DP (mmHg)	83.5 ± 12.04	106 ± 13.28	<.0001
WC (cm)	87.5 ± 9.27	107.9 ± 11.43	<.0001
BMI (kg/m^2^)	25.5 ± 5.19	36.58 ± 5.39	<.0001
HDL-C (mmol/L)	1.25 ± 0.19	0.99 ± 0.19	<.0001
LDL-C (mmol/L)	2.81 ± 0.57	3.85 ± 0.63	<.0001
FG (mmol/L)	5.31 ± 0.6	6.07 ± 0.69	<.0001
FIns (*μ*U/mL)	10.82 ± 4.99	29.58 ± 7.96	<.0001
Tryg (mmol/L)	1.86 ± 0.42	2.46 ± 0.42	<.0001
TC (mmol/L)	4.63 ± 0.64	5.64 ± 0.65	<.0001
MetS *n* (%)	163 (24.7)	199 (94.8)	<.0001
AH *n* (%)	377 (57.2)	180 (85.7)	<.0001
Dyslipidemia *n* (%)	313 (47.5)	205 (98)	<.0001

Data are expressed as mean ± SD.

The Mann-Whitney *U* test was used to compare the data between insulin sensitive and insulin resistant.

Chi-square test was used to compare the prevalence of MetS, AH, and dyslipidemia between insulin sensitive and insulin resistant.

ApoB/ApoA-I ratio: apolipoprotein B/apolipoprotein A-I ratio, ApoA-I: apolipoprotein A-I, ApoB: apolipoprotein B, SP: systolic blood pressure, DP: diastolic blood pressure, WC: waist circumference, BMI: body mass index, HDL-C: high-density lipoprotein cholesterol, LDL-C: low-density lipoprotein cholesterol, FG: fasting glucose, FIns: fasting insulin, TG: triglyceride, TC: total cholesterol, HOMA2-IR: homeostasis model assessment of insulin resistance, MetS: metabolic syndrome, and AH: arterial hypertension.

Insulin sensitive (defined as the gender-specific first three quartiles) HOMA2-IR (homeostasis model assessment of insulin resistance) <2.9; insulin resistant (defined as the gender-specific upper quartile) HOMA2-IR (homeostasis model assessment of insulin resistance) ≥2.9.

**Table 4:** Comparison of ApoB and ApoB/ApoA-I ratio with LDL-C among HOMA2-IR quartiles in women and men.

Gender	Quartiles	Subjects *n* (%)	ApoB	ApoB/ApoA-I ratio	LDL-C
Female	1 (<1.0)	163 (25)	0.48	0.52	2.33
	2 (1.0–1.29)	162 (24.8)	0.71	0.62	2.64
	3 (1.30–2.29)	166 (25.4)	0.77	0.71	2.89
	4 (≥2.30)	162 (24.8)	1.01	1	3.09

Male	1 (<1.10)	218 (25.1)	0.64	0.66	2.45
	2 (1.10–1.49)	216 (24.9)	0.84	0.76	2.82
	3 (1.50–2.89)	225 (25.8)	0.87	0.86	3.16
	4 (≥2.90)	210 (24.2)	1.11	1.12	3.85

Quartiles: HOMA2-IR quartiles with ranges, ApoB: apolipoprotein B, ApoB/ApoA-I ratio: apolipoprotein B/apolipoprotein A-I ratio, and HDL-C: high-density lipoprotein cholesterol.

**Table 5:** Logistic regression models predicting insulin resistance.

*n* = 1522	B	Standard error	Wald.	*P* value	OR	95% CI for OR	
						Lower	Upper
Gender (1)	2.137	.251	73.549	<.0001	8.641	5.279	14.146
Age	.031	.006	29.592	<.0001	1.032	1.020	1.043
ApoB/ApoA-I ratio	2.888	.412	49.187	<.0001	17.95	8.011	40.24
DP	.060	.009	48.439	<.0001	1.062	1.044	1.080
HDL-C	−2.072	.542	14.599	<.0001	.126	.044	.365
LDL-C	.155	.184	.711	.048	1.168	.814	1.675
FG	1.321	.146	81.463	<.0001	3.749	2.813	4.994
TG	.829	.263	9.953	.002	2.290	1.369	3.833

Nagelkerke *R* ^2^ of the model: 0.693.

OR: odds ratio, CI: confidence interval, ApoB/ApoA-I ratio: apolipoprotein B/apolipoprotein A-I ratio, SP: systolic blood pressure, DP: diastolic blood pressure, WC: waist circumference, HDL-C: high-density lipoprotein cholesterol, LDL-C: low-density lipoprotein cholesterol, FG: fasting glucose, TG: triglyceride, and TC: total cholesterol.

**Table 6:** Multiple regression models predicting insulin resistance.

Predictors	Unstandardized coefficients		Standardized coefficients	*t*	*R* ^ 2^ change	Significance
	*B *	Standard error	Beta			
ApoB/ApoA-I ratio	1.448	.099	.305	14.701	.421	<.0001
FG	.496	.031	.300	15.846	.133	<.0001
DP	.019	.002	.225	9.321	.039	<.0001
Gender	−.460	.045	−.176	−10.287	.026	<.0001
LDL-C	.250	.045	.128	5.519	.014	<.0001
Age	.005	.001	.062	3.929	.004	<.0001
TG	.199	.067	.067	2.954	.002	.003

*R* ^2^change is calculated by comparing *R* ^2^ values obtained from each model.

Coefficients, *t*, and *P* values are shown for final model.

Model 1: predictors—ApoB/ApoA-I ratio.

Model 2: predictors—ApoB/ApoA-I ratio and fasting glucose (FG).

Model 3: predictors—ApoB/ApoA-I ratio, FG, and diastolic pressure (DP).

Model 4: predictors—ApoB/ApoA-I ratio, FG, DP, and gender.

Model 5: predictors—ApoB/ApoA-I ratio, FG, DP, gender, and low-density lipoprotein cholesterol (LDL-C).

Model 6: predictors—ApoB/ApoA-I ratio, FG, DP, gender, LDL-C, and age.

Model 7: predictors—ApoB/ApoA-I ratio, FG, DP, gender, LDL-C, age, and triglycerides (TG).
